# Exome Sequencing of an Adult Pituitary Atypical Teratoid Rhabdoid Tumor

**DOI:** 10.3389/fonc.2015.00236

**Published:** 2015-10-23

**Authors:** Swethajit Biswas, Madeleine Wood, Abhijit Joshi, Nick Bown, Lisa Strain, Tommy Martinsson, James Campbell, Alan Ashworth, Amanda Swain

**Affiliations:** ^1^Northern Institute for Cancer Research (NICR), Newcastle University Medical School, Newcastle-upon-Tyne, UK; ^2^Northern Centre for Cancer Care (NCCC), Freeman Hospital, Newcastle University Teaching Hospitals NHS Foundation Trust, Newcastle-upon-Tyne, UK; ^3^Department of Neuropathology, Royal Victoria Infirmary (RVI), Newcastle University Teaching Hospitals NHS Foundation Trust, Newcastle-upon-Tyne, UK; ^4^International Centre for Life, Institute of Human Genetics, Newcastle University, Newcastle-upon-Tyne, UK; ^5^Department of Medical Genetics, University of Gothenburg, Gothenburg, Sweden; ^6^Institute of Cancer Research (ICR), London, UK

**Keywords:** exome sequencing, atypical teratoid rhabdoid tumor, copy number variation, adult, *SMARCB1*, trisomy 8

## Abstract

Atypical teratoid rhabdoid tumors (AT/RTs) are rare pediatric brain tumors characterized by bialleic loss of the *SMARCB1* tumor suppressor gene. In contrast to pediatric AT/RT that has a simple genome, very little is known about the adult AT/RT genomic landscape. Using a combination of whole-exome sequencing and high-resolution SNP array in a single adult pituitary AT/RT, we identified a total of 47 non-synonymous mutations, of which 20 were predicted to cause non-conservative amino acid substitutions, in addition to a subclone of cells with trisomy 8. We suggest that adult AT/RT may not be markedly dissimilar to other adult brain tumors where mutations in a range of genes, reflecting the functional specialization of different brain regions, but including *SMARCB1* inactivation, may be required for its pathogenesis.

## Introduction

Atypical teratoid rhabdoid tumors (AT/RTs) are rare and aggressive pediatric malignant rhabdoid tumors (MRT) that occur within the brain. AT/RTs are characterized by biallelic loss of *SMARCB1*, a tumor suppressor gene on chromosome 22q11.23, which encodes INI-1. INI-1 is a canonical non-catalytic component of multimeric chromatin remodeling complexes (1, 2) that act as transrepressors for proto-oncogenes, such as *c-MYC* (3). Investigations using a combination of whole-exome sequencing (WES) and high-resolution (hr)-SNP arrays have demonstrated that the untreated pediatric primary AT/RT genome is remarkably simple with very few detectable somatic mutations or copy number variations (4).

Atypical teratoid rhabdoid tumor is an extremely rare malignancy in adults with only 42 cases having been reported to date, of which 8 cases, all in female patients, have arisen from the pituitary (5, 6). Here for the first time, we report the findings of WES on a primary pituitary AT/RT from a 48-year-old peri-menopausal female.

The patient had initially presented to the referring neurosurgical center with a 2-week history of visual field disturbance. An initial contrast CT brain scan demonstrated an enhancing pituitary mass, and this was deemed to have malignant characteristics on a contrast MRI scan. Transsphenoidal pituitary surgery was performed to achieve immediate debulking and tissue material was sent for pathological analysis. This procedure was deemed to have been a gross tumor resection on post-operative MRI. The patient was discharged without complication and had achieved an almost complete symptomatic response in her presenting visual symptoms. Preliminary histopathology review at the referring center diagnosed this tumor as a “small round blue cell tumor,” but no surplus tissue material was available for further investigations.

The patient was re-admitted 2 weeks later after her initial surgery with an abrupt recurrence of her original visual field symptoms. A repeat MRI scan confirmed recurrence of the pituitary mass. Given the rapid tempo of re-growth, the neurosurgical team at the referring center performed a repeat gross tumor resection procedure where additional, multiple, tissue samples were sent for histopathology. Given the atypical preliminary diagnosis from the first tumor resection, tissue material from this second surgery was sent to our reference pediatric brain tumor pathology laboratory. Sufficient surplus tissue material was available on this occasion for additional immunohistochemical and genetic investigations to be performed at our center. The patient represented to the referring center 10 days after her second surgery with further neurological symptoms that were confirmed on MRI to have been caused by a second local post-surgical recurrence.

Second pathological review at our center found the presence of rhabdoid cells in over 90% of the tumor material (Figure S1A in Supplementary Material) and negative immunostaining for INI-1 within the tumor except for intratumoral blood vessels (Figure S1B in Supplementary Material). Although genetic confirmation of *SMARCB1* inactivation was still pending, the pathological diagnosis was compelling for AT/RT since the predominant cellular morphology was rhabdoid in nature with negative INI-1 immunostaining. Given these pathological findings, and the clinically aggressive natural history of this tumor that was refractory to two serial gross tumor resection procedures performed over a period of only 2 weeks, the patient was transferred to our oncology center, where she immediately commenced a multi-agent, non-cross resistant, AT/RT chemotherapy regimen. This chemotherapy regimen consisted of six planned cycles of vincristine, doxorubicin, and cyclophosphamide (VDC) alternating with ifosfamide, carboplatin, and etoposide (ICE) which was to be followed by pituitary and cranio-spinal irradiation. The patient refused intrathecal methotrexate treatment in-between systemic chemotherapy cycles.

A mid-treatment MRI scan after administration of three cycles of chemotherapy demonstrated a complete radiological response in the sella turcica with no evidence of neuro-axis metastatic disease. However, she was re-admitted 2 weeks after this mid-treatment MRI scan, and immediately prior to her fourth cycle of chemotherapy, with progressively worsening neurological signs. A repeat MRI brain scan diagnosed leptomeningeal disease, without recurrence of the primary tumor, and she died 1 week later (Figures S2A,B in Supplementary Material). Her rapidly deteriorating neurological condition during this admission precluded a diagnostic lumbar puncture being performed for cerebrospinal fluid cytological/molecular pathological analysis.

## Methods

This study was carried out in accordance with the recommendations of the Medical Research Ethical Committee (MREC*)* in Newcastle-Upon-Tyne, UK. Tissue was stored in accordance with local ethical approval for biobanking malignant tissue from deceased patients (MREC: 10/H1306/50 – approval 2011).

Details about WES and SNP array methodology, data analysis are provided in a Supplementary Appendix.

## Results

**Figure 1:**
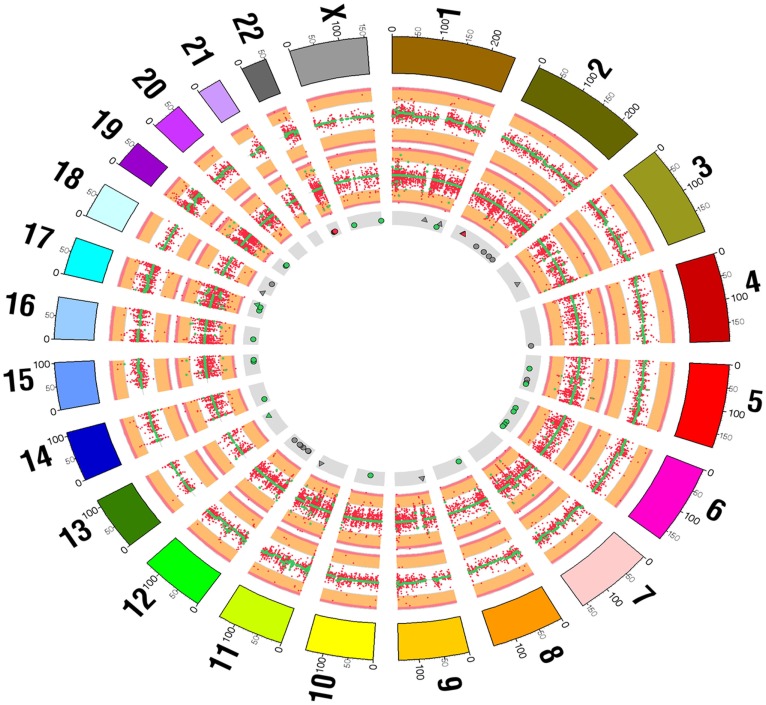
**Summary of exome sequencing results comparing tumor and control skin tissue displayed as a Circos diagram**. The outermost ring indicates chromosomes. The next ring summarizes DNA copy number and shows a scatter plot of the log2 ratio of normalized DNA concentration (RPKM) in the tumor and normal samples (red points). The green points indicate the inferred segmented copy number profile. The third ring from the outermost summarizes variant allele frequencies across the chromosomes (red points) and segmented allele frequencies (green points). The innermost ring indicates the positions of somatic SNP (circles) and indels (triangles). Somatic mutations likely to lead to a change in an encoded protein are colored green and those that are also included in the Cancer Gene Census list are colored red. All other somatic mutations are colored gray.

Whole-exome sequencing revealed a total of 98 somatic mutations. The overall mutation rate was 1.89 per Mb of exome sequence (Figure 1). Of the identified 98 mutations, 88 were single nucleotide substitutions and 10 were indels. Of the 88 single nucleotide substitutions, 56 were transitions and 32 were transversions. Of the 98 mutations detected, 49 were predicted to alter the sequence of the encoded protein via missense, nonsense, or frameshift mutations (Table S1 in Supplementary Material).

Two of these 49 mutations were distinct biallelic mutations in *SMARCB1* at Exon 2 codon 49 (Ser49) C → G, which is predicted to cause a stop codon, and at Exon 5 codon 628 + 2 T → G, which is predicted to cause alternative splicing. Both *SMARCB1* mutations were present in the Cancer Gene Census List^1^. A non-synonymous mutation in the mismatch repair (MMR) gene *MSH2* was also present (see Table S1 in Supplementary Material). However, inactivating mutations in *MSH2* are known to cause microsatellite instability, which tends to result in a substantially higher number of somatic mutations (>1000 per exome) than we have detected. Therefore, the identified *MSH2* mutation seems unlikely to have led to loss of MMR activity.

Apart from the two separate *SMARCB1* mutations, *in silico* bioinformatic analysis using POLYPHEN2^2^ predicted that 20/47 of the remaining non-synonymous mutations could have caused non-conservative amino acid substitutions that would have been detrimental to protein function. Nineteen of these non-synonymous mutations were novel in the COSMIC database. We categorized these 20 mutated genes into four functionally distinct groups based upon wild-type gene function (GeneDistiller 2): Group 1: intracellular transport/cytoskeleton – tubulin and actin dynamics genes; Group 2: stem cell differentiation genes; Group 3: cell metabolism genes; Group 4: cytotoxic resistance genes (Table 1).

**Table 1:** **Twenty non-synonymous mutations predicted by POLYPHEN2 to be detrimental to cognate protein function**.

Functional groups	Gene	Novel to COSMIC database (Yes (Y); No (N))
Group 1: tubulin/actin transport genes	C16orf70	Y
	C13orf25	Y
	MEI1	Y
	LRRCC1	Y
	TTLL4	Y
	JAKMIP1	Y
	MCC	Y
	CTNNA3	Y
	MYO5A	Y
Group 2: stem cell differentiation genes	PARD3B	Y
	TIAM2	Y
	T	Y
	LNX1	Y
Group 3: metabolism genes	PYGL	N
	MTO1	Y
	POFUT1	Y
	SLC25A1	Y
	CYP1A1	Y
Group 4: cytotoxic resistance genes	GSTA4	Y
	APAF1	Y

We used hrSNP array on the fresh-frozen tumor sample to analyze the tumor for copy number variation and ploidy. We identified a subclone of cells with isolated trisomy 8, which using a *C-MYC* FISH probe, was confirmed to be present in 11% of AT/RT cells (Figure S3 in Supplementary Material). We were unable to investigate the mutational background of these +8 cells because of a lack of surplus tissue.

## Discussion

The genetic analysis of this single adult AT/RT case, using a combination of WES and hrSNP array, has demonstrated a greater level of genomic complexity than that has been previously recognized in children. We found a higher mutation rate compared to the mean mutation rate of 0.19 per Mb of exome sequence demonstrated in a series of 32 pediatric primary AT/RTs examined by Lee et al. (4). Our own findings are also distinct from those recently reported by Wu et al. in an adult superior colliculus AT/RT that demonstrated only four mutations on a background of *SMARCB1* inactivation, which would be comparable to the low background mutational frequency in pediatric AT/RTs. These mutations were in *MDM4*, *RHPN2*, *FLT3*, and *NPRL3*, but were not detected in our own patient’s tumor (7).

Moreover, our finding of trisomy 8 in a minority of AT/RT cells would be consistent with the concept of intratumoral heterogeneity that has already been demonstrated at the mutational level in extracranial primary solid tumors (8, 9). In addition to containing the locus for the *c-MYC* proto-oncogene, chromosome 8 also harbors loci for a number of other genes that have been implicated in the development of tumor progression, such as cancer cell migration (i.e., MT3-MMP), cell survival (i.e., BNIP3), and dedifferentiation (i.e., Oct-4)^3^. However, in the absence of cerebrospinal fluid cells for analysis from this patient at intra-chemotherapy relapse, we were unable to confirm whether the +8 subclone was causative in this patient’s leptomeningeal disease progression.

In summary, we have identified a number of novel mutations involving functionally unique genes, as well as copy number variation heterogeneity affecting chromosome 8 in an adult AT/RT, but we are unable to establish the phenotypic effect of these changes without performing functional studies. Similarly, we cannot prove at this stage, without analysis of a larger set of adult AT/RTs, that the mutations we have detected were specific to the pituitary site of origin.

However, in combination, the adult AT/RT exome sequence findings presented here, those of Wu et al. (7), and the pediatric AT/RT transcriptomic data from Torchia et al. (10), would be consistent with the hypothesis that the genomic landscape of a primary AT/RT is dependent upon the neuroanatomical site in which it arises. This might be a reflection of the regionally specified neural stem cell lineage from which the tumor arose.

We suggest that future studies in AT/RT should take an extended integrative genomic approach by using next-generation sequencing in combination with a variety of different tumor “-omic” techniques, including those used by Torchia et al. to identify different prognostic sub-populations based upon a combination of transcriptomics and immunohistochemistry (10). This approach might enable the identification of patient sub-populations that harbor potentially actionable mutations that are associated with a poor prognosis.

We also advocate a systematic genetic comparison of adult and pediatric AT/RTs in future multinational studies, since loss of *SMARCB1* within a biologically specific developmental window within neuronal stem cells *in utero* may be sufficient in itself to trigger AT/RT formation in babies and infants (4, 11). However, additional and co-operative non-synonymous mutations in the post-natal setting may be required for the development of AT/RT in adults, similar to the paradigm in pediatric ALL where additional genomic anomalies are required for the pathogenesis of *MLL* rearranged B-cell ALL in older children (11).

In this regard, our findings of potentially functionally detrimental mutations in a number of genes involved in stem cell differentiation, such as *LNX1* (modulation of NOTCH1 signaling), *TIAM2* (regulation of neurite outgrowth), *PARD3B* (regulation of cell polarity), and *T* (an initiator of neuronal stem cell differentiation) might be hypothesis forming in that prevention of stem cell differentiation, therefore “locking-in” cells into the stem cell progenitor pool, may be required for effective *SMARCB1*-mediated carcinogenesis in post-natal neuronal stem cells. This would facilitate self-renewal of neuronal stem cells without any stem cells being able to escape the phenotypic effects of *SMARCB1* inactivation by undergoing differentiation. This hypothesis awaits confirmation, but we believe that the genetic findings presented within this paper, in combination with the exome sequencing data from Wu et al. (7), suggest that the development of AT/RT in adults may not be exclusively dependent upon *SMARCB1* inactivation, such that adult AT/RT may not be markedly dissimilar to other adult brain tumors where co-operative mutations in a range of genes, reflecting the functional specialization of different brain regions, may be required for its pathogenesis.

## Conflict of Interest Statement

The authors declare that the research was conducted in the absence of any commercial or financial relationships that could be construed as a potential conflict of interest.

## Footnotes

^1^http://cancer.sanger.ac.uk/cancergenome/projects/census/

^2^http://genetics.bwh.harvard.edu/pph2/

^3^http://atlasgeneticsoncology.org/Indexbychrom/idxa_8.html
