# Supplementary material for: Exome Sequencing of an Adult Pituitary Atypical Teratoid Rhabdoid Tumor
Source: Front Oncol. 2015 Oct 23;5:236. doi: 10.3389/fonc.2015.00236 (PMC4617150; doi:10.3389/fonc.2015.00236)
Supplement: Supplementary file 1 [file Table_1.PDF]

Supplementary Table 1

Gene	Mutation (GRCh37)	Transcript	Protein change	Mutation consequence
SMARCB1	22:24145611 (T>G)	ENST00000407422	-	Essential Splice Site
SMARCB1	22:24133995 (C>G)	ENST00000407422	p.S49*	Stop Gained
MSH2	2:47641559-47641560 (GTA>G)	ENST00000406134	-	Splice Site, Essential Splice Site, Intronic
JAKMIP1	4:6086623 (G>A)	ENST00000282924	p.R302W	Non-Synonymous Coding
GPAT2	2:96688929 (G>A)	ENST00000359548	p.R692C	Non-Synonymous Coding
PARD3B	2:206305242 (A>G)	ENST00000358768	p.T902A	Non-Synonymous Coding
POFUT1	20:30803126 (C>T)	ENST00000375730	p.R101W	Non-Synonymous Coding
C16orf70	16:67165209 (G>T)	ENST00000219139	p.L84F	Non-Synonymous Coding
C13orf26	13:31540530 (G>A)	ENST00000380473	p.G214D	Non-Synonymous Coding
IL22RA2	6:137476236 (T>C)	ENST00000339602	p.K73R	Non-Synonymous Coding
T	6:166571844 (G>A)	ENST00000366871	p.L365F	Non-Synonymous Coding
PSD3	8:18662349 (A>C)	ENST00000523619	p.I500S	Non-Synonymous Coding
OR4M1	14:20249295 (G>A)	ENST00000315957	p.V272M	Non-Synonymous Coding
SNF8	17:47018378 (G>A)	ENST00000290330	p.A51V	Non-Synonymous Coding
PLXNB3	X:153035555 (G>T)	ENST00000538	p.L96F	Non-Synonymous

		543		Coding
PYGL	14:51387281 (C>T)	ENST00000216392	p.R278Q	Non-Synonymous Coding
HERC1	15:64005069 (G>A)	ENST00000425434	p.R481W	Non-Synonymous Coding
LNX1	4:54374341 (T>A)	ENST00000306888	p.Q49L	Non-Synonymous Coding
ZNF300	5:150275461 (C>A)	ENST00000418587	p.S411I	Non-Synonymous Coding
EFCAB8	20:31481024 (G>A)	ENST00000400522	p.G276D	Non-Synonymous Coding
RNFT1	17:58034688 (G>A)	ENST00000305783	p.P301L	Non-Synonymous Coding
MEI1	22:42112103 (T>A)	ENST00000401548	p.L161*	Stop Gained
KRT33A	17:39502830 (A>T)	ENST00000007735	p.S323T	Non-Synonymous Coding
GSTA4	6:52843315 (G>C)	ENST00000370960	p.P115A	Non-Synonymous Coding
MYO5A	15:52668566 (C>T)	ENST00000546028	p.V430M	Non-Synonymous Coding
ZFHX3	16:72821323 (G>A)	ENST00000397992	p.P2704S	Non-Synonymous Coding
APAF1	12:99042195 (A>T)	ENST00000357310	p.I20F	Non-Synonymous Coding
CNKSR2	X:21534649 (C>A)	ENST00000379510	p.P286Q	Non-Synonymous Coding
MCC	5:112458485 (C>T)	ENST00000302475	p.S118N	Non-Synonymous Coding
CTNNA3	10:67680132 (G>C)	ENST00000433211	p.P882A	Non-Synonymous Coding
MEI1	22:42112102 (T>A)	ENST00000401	p.L161	Non-Synonymous

		548	M	Coding
LRRCC1	8:86042295 (T>A)	ENST00000426019	-	Essential Splice Site
BAZ2B	2:160287434 (A>G)	ENST00000355831	p.S712P	Non-Synonymous Coding
TTLL4	2:219616455 (C>T)	ENST00000258398	p.R968*	Stop Gained
CENPH	5:68490522 (C>T)	ENST00000515001	p.A80V	Non-Synonymous Coding, Splice Site
RNH1	11:498031 (G>A)	ENST00000397614	p.A356V	Non-Synonymous Coding
CYP1A1	15:75015179 (C>T)	ENST00000379727	p.S87N	Non-Synonymous Coding
SLC35A1	6:88210383 (C>A)	ENST00000369556	p.Q118K	Non-Synonymous Coding, Splice Site
VAV3	1:108299932 (G>A)	ENST00000371846	p.T281I	Non-Synonymous Coding
TRIM49L2	11:89774252 (G>A)	ENST00000448984	p.S298N	Non-Synonymous Coding
TIAM2	6:155465853 (C>T)	ENST00000461783	p.P582S	Non-Synonymous Coding
USH2A	1:215960060 (C>A)	ENST00000366943	p.A3447S	Non-Synonymous Coding
DHX34	19:47858461 (C>T)	ENST00000466298	p.L291F	NMD Transcript, Non-Synonymous Coding
LNX1	4:54374340 (T>A)	ENST00000306888	p.Q49H	Non-Synonymous Coding
PSAT1	9:80923424 (G>A)	ENST00000421149	p.R46Q	Non-Synonymous Coding
MTO1	6:74207588 (G>C)	ENST00000370300	p.R654P	Non-Synonymous Coding
				Non-Synonymous

KIRREL3	11:126314973 (G>A)	ENST00000529097	p.L385F	Coding
ABCA8	17:66928555 (CA>C)	ENST00000269080	p.-224	Frameshift Coding
ZIC2	13:100635008-100635010 (CCCA>C)	ENST00000425702	p.AH230-231A	Non-Synonymous Coding
FANCD2	3:10105439 (A>C)	ENST00000421731	-	NMD Transcript, Intronic
-	17:59147356 (C>A)	ENST00000437246	-	Within Non Coding Gene
C6orf183	6:109517804 (A>T)	ENST00000417143	-	Splice Site, Within Non Coding Gene, Intronic
MAP2K6	17:67513721 (G>T)	ENST00000359094	-	Synonymous Coding
PRAMEF2	1:12919173 (A>G)	ENST00000240189	-	Intronic
-	14:50298906 (C>A)	ENST00000358799	-	Downstream
-	17:59147568 (G>C)	ENST00000437246	-	Within Non Coding Gene
MEGF10	5:126755818 (C>T)	ENST00000508365	-	Synonymous Coding
E2F7	12:77439831 (G>A)	ENST00000552907	-	NMD Transcript, Synonymous Coding
TXLNB	6:139568975 (C>T)	ENST00000358430	-	Synonymous Coding
TTN	2:179476639 (C>T)	ENST00000359218	-	Synonymous Coding
ERBB4	2:213291118 (C>T)	ENST00000484594	-	Within Non Coding Gene, Intronic
ANKRD36	2:97877251 (A>G)	ENST00000421	-	Upstream

		946		
PRSS1	7:142459667 (G>A)	ENST00000486171	-	Synonymous Coding
DNAH2	17:7669791 (C>T)	ENST00000360606	-	Synonymous Coding
DLAT	11:111930794 (G>A)	ENST00000280346	-	Splice Site, Intronic
BIRC6	2:32733162 (C>T)	ENST00000421745	-	Synonymous Coding
PAK2	3:196529926 (C>A)	ENST00000327134	-	Synonymous Coding
VWFP1	22:17178056 (T>G)	ENST00000457911	-	Within Non Coding Gene
WDR43	2:29145867 (C>G)	ENST00000466067	-	Upstream
RBM27	5:145598593 (G>A)	ENST00000265271	-	Synonymous Coding
CCDC28A	6:139109556 (G>T)	ENST00000026464	-	Intronic
SVOP	12:109306401 (C>T)	ENST00000299134	-	Synonymous Coding
RUNX2	6:45514673 (T>C)	ENST00000359524	-	Synonymous Coding
OBP2A	9:138441125 (A>C)	ENST00000471886	-	NMD Transcript, Intronic
NEK3	13:52707991 (G>T)	ENST00000258597	-	NMD Transcript, Intronic
SYNJ2	6:158438227 (C>T)	ENST00000449859	-	Upstream
-	14:22070165 (C>T)	ENST00000312058	-	Within Non Coding Gene
SNX27	1:151665092 (G>T)	ENST00000368841	-	NMD Transcript, Intronic

CNPY3	6:42892022 (C>T)	ENST00000394142	-	Upstream
-	2:87085143 (A>G)	ENST00000441646	-	Upstream
LRRCC1	8:86042299 (T>A)	ENST00000426019	-	Splice Site, Intronic
KRT25	17:38910275 (G>A)	ENST00000312150	-	Splice Site, Intronic
PPP1R12A	12:80202369 (G>A)	ENST00000550001	-	Within Non Coding Gene, Intronic
GIMAP6	7:150327137 (T>A)	ENST00000392862	-	5' UTR
ARHGAP23	17:36654080 (C>T)	ENST00000547158	-	Within Non Coding Gene, Intronic
PCNT	21:47768979 (C>A)	ENST00000359568	-	Synonymous Coding
LYRM5	12:25362903 (G>C)	ENST00000381356	-	Downstream
PCDHB9	5:140568811 (G>C)	ENST00000316105	-	Within Non Coding Gene
ZNF280B	22:22842518 (G>A)	ENST00000406426	-	Synonymous Coding
POLR2J4	7:44032610 (A>G)	ENST00000422304	-	Within Non Coding Gene, Intronic
ZDHHC11	5:840704 (G>A)	ENST00000507800	-	NMD Transcript, Synonymous Coding
PLEKHH2	2:43931081-43931082 (TGC>T)	ENST00000405000	-	Within Non Coding Gene, Intronic
EI24	11:125442504 (CT>C)	ENST00000527235	-	NMD Transcript, Intronic
ENAH	1:225700349 (TA>T)	ENST00000488523	-	Within Non Coding Gene, Intronic

SPICE1	3:113169373 (GA>G)	ENST00000496105	-	Within Non Coding Gene, Intronic
-	18:49103-49104 (C>CGGTGGGAGAAGGACGGGGGTCTCACCGCTGGCCTCGTGGTGGTGCACGTTG)	ENST00000408514	-	Downstream
SNX27	1:151666827 (CT>C)	ENST00000368841	-	NMD Transcript, 3' UTR
FREM1	9:14824709-14824710 (C>CTA)	ENST00000380875	-	NMD Transcript, Intronic

Supplementary Table I. Somatic mutations detected in the pituitary AT / RT tumor. Orange shaded rows show mutations annotated in the Cancer Gene Census List (http://cancer.sanger.ac.uk/cancergenome/projects/census/). Yellow shaded rows show an additional 20 nonsynonymous mutations indentified by POLYPHEN2 as being potentially detrimental to protein function, of which all except for *PYGL*, were novel.
