# Supplementary material for: Exome Sequencing of an Adult Pituitary Atypical Teratoid Rhabdoid Tumor
Source: Front Oncol. 2015 Oct 23;5:236. doi: 10.3389/fonc.2015.00236 (PMC4617150; doi:10.3389/fonc.2015.00236)
Supplement: Supplementary file 2 [file Data_Sheet_1.PDF]

Supplementary Appendix

Methods

Whole-Exome Sequencing (WES)

Description of sequencing and file generation

DNA samples were extracted from a formalin-fixed paraffin embedded (FFPE) tumor sample which represented almost all of the remaining tissue material obtained at the patient's first attempted GTR. FFPE tissue from the patient's left leg skin biopsy was used as a control. Targeted exome capture was performed using SureSelect Human All Exome v3 reagents (Agilent). Illumina paired-end libraries were prepared from the captured target regions and sequenced on a HiSeq2000 (Illumina) acquiring 2 x 76 bp reads. Casava software (v1.8, Illumina) was used to make base calls and demultiplex the sequencing data. Sequences were output in fastq format. Reads failing the Illumina chastity filter were removed before further analysis.

Sequence alignment and detection of candidate mutations

BWA was used to align reads to the human reference genome (GRCh37). PCR duplicates were removed prior to further processing and variant detection. Variant calling was done using GATK Broad Best pipeline V2 with standard settings. Variants called in regions not covered by the capture probes were excluded, as were those with genotype qualities below 20 and those covered by fewer than 10 reads in either sample.

Somatic indel mutations were selected as those variants detected with allele fractions of 15% or greater in the tumor sample than that were present at 5% or less in the control skin sample. Somatic mutations were identified using a Bayesian methodology with Mutect software coupled with filters that provide high specificity.

ASCAT software was used to determine tumour purity and allele-specific ploidy. An equivalent to LogR values were generated as the median-centered log2 ratio of RPKM values calculated from the tumour and normal exome sequencing data. The LogR of the normal sample was set to zero. Equivalents to BAF values were obtained from the proportion of reads reporting each variant out of the total number of reads covering each locus, i.e. the variant allele fractions for the tumor and normal samples. Regions that had undergone loss-of-heterozygosity (LOH) were selected as those segmented regions of the exome where the ploidy of germline heterozygous alleles was zero in the tumor sample. All findings were represented as a Circos diagram. The novelty, or otherwise, of identified nonsynonmous somatic mutations, which were more likely to lead to non-conservative amino acid substitutions of the respective cognate protein, were cross-referenced to the Catalogue of Somatic Mutations in Cancer (COSMIC) database (http://sanger.ac.uk//cosmic)

Polyphen2 Software Programme for Prediction of Putative Phenotypically Compromising Mutations

The predicted functional phenotype of the identified mutations were analysed using the POLYPHEN2 software tool (http://genetics.bwh.harvard.edu/pph2/)

High-Resolution SNP Array

High-resolution SNP array was performed as previously described using Illumina Technology™. Specific methodology details can be requested from Professor Tommy Martinsson at Gothenburg University, Sweden; email: tommy.martinsson@clingen.gu.se
