# Supplementary material for: Exome Sequencing of an Adult Pituitary Atypical Teratoid Rhabdoid Tumor
Source: Front Oncol. 2015 Oct 23;5:236. doi: 10.3389/fonc.2015.00236 (PMC4617150; doi:10.3389/fonc.2015.00236)
Supplement: Supplementary file 3 [file Image_1.PDF]

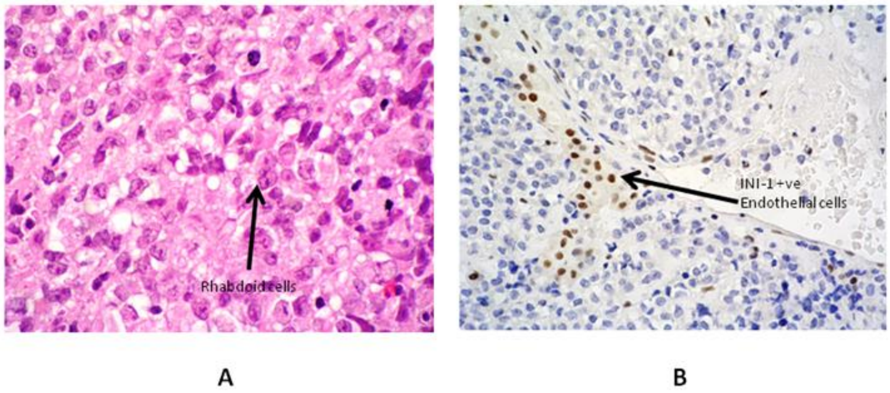

Figure 1. H&E photomicrograph.

A. H&E staining of pituitary AT / RT demonstrating rhabdoid cells (arrow)

B. INI-1 negatively staining nuclei in AT / RT cells, but INI-1 positive nuclear staining within adjacent (positive control) endothelial cells (arrow).
