# Supplementary material for: Exome Sequencing of an Adult Pituitary Atypical Teratoid Rhabdoid Tumor
Source: Front Oncol. 2015 Oct 23;5:236. doi: 10.3389/fonc.2015.00236 (PMC4617150; doi:10.3389/fonc.2015.00236)
Supplement: Supplementary file 4 [file Image_2.PDF]

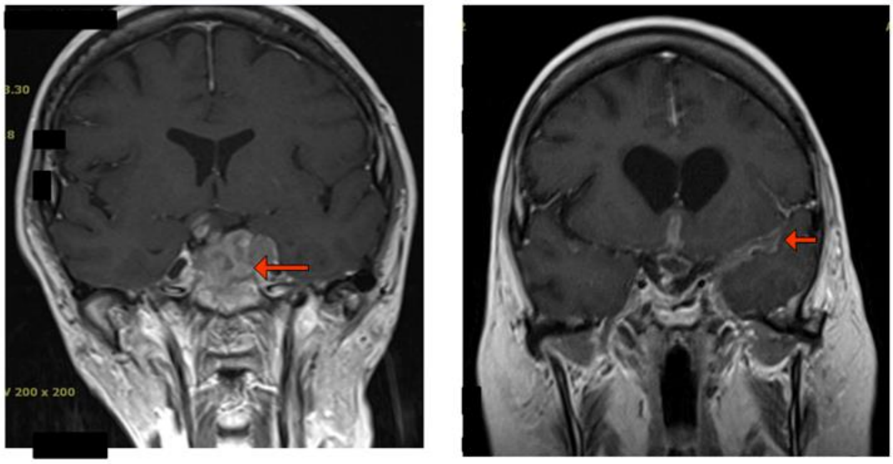

A B

Figure 2.

A. Pre-chemotherapy contrast-enhanced T1-weighted coronal MRI brain scan (arrow – large enhancing pituitary mass replacing the entire gland)

B. Intracranial disease progression; contrast-enhanced T1-weighted coronal MRI brain scan demonstrating left supra-temporal fossa leptomenigeal metastasis (arrow) *after* the 3rd cycle of chemotherapy. Note the almost complete radiological response within the sella turcica.
