# Supplementary figures and images for: Exome Sequencing of an Adult Pituitary Atypical Teratoid Rhabdoid Tumor
Source: Front Oncol. 2015 Oct 23;5:236. doi: 10.3389/fonc.2015.00236 (PMC4617150; doi:10.3389/fonc.2015.00236)

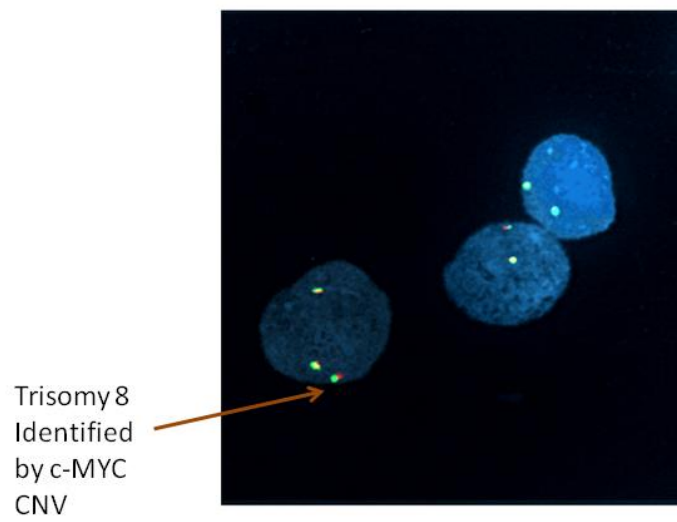

**Figure 3.**

FISH for c-MYC demonstrating solitary trisomy 8 (demonstrable in 11% of AT / RT cells)

Supplement: Supplementary file 5 [file Image_3.PDF]
